# Preoperative systemic immune-inflammation index predicts prognosis of patients with oral squamous cell carcinoma after curative resection

**DOI:** 10.1186/s12967-018-1742-x

**Published:** 2018-12-18

**Authors:** Pengfei Diao, Yaping Wu, Jin Li, Wei Zhang, Rong Huang, Chen Zhou, Yanling Wang, Jie Cheng

**Affiliations:** 1 Jiangsu Key Laboratory of Oral Disease, Nanjing Medical University, 136 Hanzhong Road, Nanjing, 210029 Jiangsu China; 2 Department of Oral and Maxillofacial Surgery, Affiliated Stomatological Hospital, Nanjing Medical University, Nanjing, 210029 Jiangsu China; 3 Department of Oral and Maxillofacial Surgery, Affiliated Hospital of Jiangnan University, Wuxi, 214000 Jiangsu China

**Keywords:** Oral squamous cell carcinoma, Prognostic biomarker, Systemic immune-inflammation index

## Abstract

**Background:**

Deregulated inflammation and immune deficit both intricately associate with cancer initiation and progression, which have been increasingly exploited as prognostic biomarkers and therapeutic targets. Recently, systemic immune-inflammation index (SII) based on peripheral neutrophil, lymphocyte and platelet counts serves as a novel and powerful cancer biomarker with prognostic significance in multiple types of malignancies. Here, we sought to evaluate the prognostic value of preoperative SII in patients with primary oral squamous cell carcinoma (OSCC) after curative resection.

**Methods:**

Two independent cohorts with a total number of 309 patients with OSCC from two tertiary referral hospitals were included and defined as training (Nanjing, 138) and validation (Wuxi, 171) cohort, respectively. Preoperative SII in both cohorts was calculated and its optimal cutoff value was initially determined by X-tile software in the training cohort and then verified in the validation cohort.

**Results:**

Our data indicated that high SII (≥ 484.5) was significantly associated with larger tumor size (*P ***< **0.05, Chi square test), reduced overall and disease-free survival (Kaplan–Meir, *P ***< **0.05, Log-rank test). Univariate and multivariate analyses further revealed that SII was an independent prognostic predictor for patient survival. Moreover, the area under receiver operating characteristic curve of SII for survival was significantly greater or comparable to other well-established prognostic parameters, indicative of its satisfactory prediction accuracy and specificity.

**Conclusions:**

Our findings reveal that high preoperative SII associates with poor outcome and serves as a non-invasive, low-cost and powerful prognostic predictor for patients with OSCC. This inflammation/immune-related biomarker holds translational potentials to supplement currently prognostic regime to better stratification of patients and treatment planning.

**Electronic supplementary material:**

The online version of this article (10.1186/s12967-018-1742-x) contains supplementary material, which is available to authorized users.

## Background

Oral cancer is characterized by a heterogonous group of epithelial malignancy arising from oral lining mucosa and overwhelmingly identified as squamous cell carcinoma (SCC) in histopathology [1]. It remains a major cause of morbidity and mortality as evidenced by several hundred thousand of new cases and deaths annually. Smoking, alcohol abuse as well as human papillomavirus (HPV) infection are common etiologic risks for oral SCC (OSCC) [2, 3]. Given oral cavity as a distinct site of the head and neck that possesses complex functional anatomy with regard to speech, swallowing, and facial esthetics, patients with OSCC inevitably suffer severe physiological deficits and social disability especially those with advanced disease following curative therapies such as surgery, chemotherapy and radiotherapy. However, the 5-year survival for patients with OSCC remains approximately 60%, although tremendous progress have been made to combat against this lethal malignancy in the past decades [2, 4]. There is a urgent need for biomarker development and validation that allow better patient stratification, treatment decision-making and prognostic prediction.

Until now, multiple clinicopathological parameters such as cervical node status and TNM stage have been established as major determinants of prognosis and treatment planning [2, 4]. However, these factors are far from optimal in partly due to imperfect performance in prognostic prediction [5]. Furthermore, to defining these parameters largely relies on postoperative histopathological examinations on clinical samples either from primary site or neck dissection, thus precluding the possibility to accurately determine these predictors prior to curative surgery. Besides those known factors associated with prognosis, additional features of patients with OSCC before therapy might be identified as preoperative biomarkers that help in guiding patients stratification, treatment decisions and prognostic prediction.

Tumor-promoting inflammation and avoiding immune destruction have been recognized as emerging hallmarks of cancer and increasingly been exploited as diagnostic and therapeutic targets with translational promise [6, 7]. Noticeably, mounting evidence has demonstrated that immune and inflammatory cells including neutrophils, platelets and lymphocytes not only drive cancer overgrowth, invasion and chemoresistance in the local tumor environment, but also facilitate metastasis by assisting tumor cell extravasation, survival in peripheral blood and subsequent reseed distant site [6, 8]. These ideas and ample evidence have led to the examinations of various immune and inflammatory markers both cancer in situ and systemic bloodstream as prognostic factors in cancer [9–13]. Multiple biomarkers including lymphocyte count, neutrophil-lymphocyte ratio (NLR), platelet–lymphocyte ratio (PLR) and C-reactive protein (CBP) have been developed and verified with prognostic values in a broad spectrum of cancer [9, 14, 15]. Recently, the systemic-immune-inflammation index (SII) based on neutrophils, lymphocytes and platelets as a jointed tool has been developed to offer prognostic information in patients with hepatocellular carcinoma, pancreatic cancer and germ-cell tumor [16–18]. This novel integrated prognostic score combining peripheral neutrophils, lymphocytes and platelets is more powerful than individual cell type-based factors in prognostic assessment, presumably due to better reflect the balance of host inflammation and immune status [19–21]. However, the prognostic value of these inflammatory and hematological markers in primary OSCC remains largely unexplored thus far.

In the present study, we sought to determine the prognostic values of the inflammation and immune-based score SII, NLR and PLR in patients with OSCC after curative resection by interrogating preoperative systemic cell counts and ratios in two independent cohorts.

## Materials and methods

### Patients

This retrospective translational study included two independent cohorts of patients with primary resectable OSCC who underwent curative resection of tumors at two tertiary referral OSCC centers. One patient set comprised 138 patients treated at the Department of Oral and Maxillofacial Surgery, Affiliated Stomatological Hospital, Nanjing Medical University from February 2006 to December 2016, which was defined as training cohort. The other set recruited 171 patients treated at Department of Oral and Maxillofacial Surgery, Affiliated Hospital of Jiangnan University between January 2004 to December 2014, which was defined as validation cohort. All patients enrolled in both training and validation cohorts were treatment-naïve before radical resection. The detailed relevant epidemiological, clinical, pathological and follow-up data were available for these eligible patients. Tumor histopathological grading and clinical staging of patients were performed according to the WHO grading system and AJCC 7th staging system, respectively [22].

Patients with active infection or inflammatory disease within 4 weeks before preoperative standard blood harvest and test were excluded. Patients with simultaneous steroid or other drugs which might affect the total amount of white blood cells were also excluded. The comparative baseline clinical characteristics of patients in training, validation and combined cohorts are described in Table 1. Written informed consent was obtained for all patients or their legal guardians. This study has been approved by the Ethic Committee of Affiliated School of Stomatology, Nanjing Medical University and Affiliated Hospital of Jiangnan University, and performed in accordance with the ethical standards of the 1964 Declaration of Helsinki.

**Table 1:** The clinicopathological characteristics of patients in the training, validation and combined cohorts

Clinical and pathological indexes	Training cohort	Validation cohort	*P*	Combined cohort
	*N *= 138 (%)	*N *= 171 (%)		*N *= 309 (%)
Age (y)				
≤ 60	47 (34.06)	65 (38.01)	0.472	112 (36.25)
> 60	91 (65.94)	106 (61.99)		197 (63.75)
Gender				
Male	81 (58.70)	90 (52.63)	0.286	171 (55.34)
Female	57 (41.30)	81 (47.37)		138 (44.66)
Smoking				
No	89 (64.49)	133 (77.78)	*0.010*	222 (71.84)
Yes	49 (35.51)	38 (22.22)		87 (28.16)
Alcohol use				
No	104 (75.36)	141 (82.46)	0.126	245 (79.29)
Yes	34 (24.64)	30 (17.54)		64 (20.71)
Tumor size				
T1–T2	103 (74.64)	135 (78.95)	0.371	238 (77.02)
T3–T4	35 (25.36)	36 (21.05)		71 (22.98)
Pathological grade				
I	83 (60.14)	95 (55.56)	0.417	178 (57.61)
II–III	55 (39.86)	76 (44.44)		131 (42.39)
Cervical nodal metastasis				
N0	112 (81.16)	121 (70.76)	*0.035*	233 (75.40)
N+	26 (18.84)	50 (29.24)		76 (24.60)
Clinical stage				
I–II	91 (65.94)	100 (58.48)	0.180	191 (61.81)
III–IV	47 (34.06)	71 (41.52)		118 (38.19)
SII				
< 484.5	96 (69.57)	105 (61.40)	0.135	201 (65.05)
≥ 484.5	42 (30.43)	66 (38.60)		108 (34.95)
NLR				
< 2.9	109 (78.99)	129 (75.44)	0.461	238 (77.02)
≥ 2.9	29 (21.01)	42 (24.56)		71 (22.98)
PLR				
< 170.2	114 (82.61)	129 (75.44)	0.126	243 (78.64)
≥ 170.2	24 (17.39)	42 (24.56)		66 (21.36)
Median survival (months)	48 (4–134)	45 (4–138)		46 (4–138)

The italic values in the table denotes *P*-values less than 0.05 with statistical significance

Patients following curative resection were routinely followed up every 3 months during the first and second postoperative year and every 6 or 12 months thereafter. Patients with cervical lymph nodes metastasis were indicated for postoperative radiotherapy. At every follow-up visit, all patients had physical examinations to monitor local recurrence and cervical metastasis. If any suspicious lesions were found, CT or MRI scan together with biopsy were performed. If local recurrence or cervical metastasis after surgery was present, second radical resection or radiotherapy/chemotherapy was given as appropriate. Radiographic surveillance (CT or MRI scan) was recommended once in each year until 5 year after surgery in patients without evidence of disease recurrence and metastasis. Overall survival (OS) was defined as the interval between initial surgery and either death or the last follow-up. Disease-free survival (DFS) was calculated as initial surgery and the presence of local recurrence, metastasis, death or the last follow-up.

### Systemic immune-inflammation index

The whole blood samples for neutrophil, monocyte and platelet counts were harvested within 3 days before surgery. As defined previously, the SII was defined as follows: SII = P × N/L, where P, N, and L were the preoperative peripheral platelet, neutrophil, and lymphocyte counts, respectively [16]. Other two ratios were calculated as follows: NLR = N/L, PLR = P/L, respectively [9, 14]. The X-tile 3.6.1 software (Yale University, New Haven, CT) was used for bioinformatics analysis of the data from training cohort to determine the cutoff value of SII, NLR and PLR for OS [23]. Hence, results from X-tile analyses revealed optimal cutoff values for SII, NLR and PLR at 484.5 × 10^9^ (indicated as 484.5 for simplicity thereafter), 2.9 and 170.2, respectively (Additional file 1: Fig. S1). The SII, NLR and PLR scores were stratified into < 484.5 or ≥ 484.5 (484.5, < 2.9 or ≥ 2.9 and < 170.2 or ≥ 170.2 as high or low subgroups for all subsequent analyses.

### Statistical analyses

Continuous variables were presented as the median and range or the mean ± SD. Analyses of the associations between SII, NLR, PLR and multiple clinicopathological parameters were conducted using Fisher’s exact test or Chi square test accordingly. The ratios of patient overall and disease-free survival were calculated with the Kaplan–Meier method and compared with log-rank test. Univariate and multivariate prognostic analyses were performed by Cox proportional hazards regression model. Receiver operating characteristics (ROC) curves were plotted to define sensitivity, specificity, and differences in the area under the curves (AUC) of indicated prognostic factors. Hazard ratios (HRs) with 95% confidence intervals (CIs) were calculated as estimates of the correlations. P values less than 0.05 were considered statistically significant. All statistical analyses were performed using GraphPad Prism 7 (GraphPad Software, La Jolla, CA) or IBM SPSS 22.0 software (SPSS Inc., Chicago, IL).

## Results

### Patients’ characteristics

A total number of 309 patients with primary resectable OSCC which met our inclusion criteria were identified and enrolled here. Among them, 138 patients were in the training cohort from Nanjing, while 171 were in the validation cohort from Wuxi. The detailed baseline characteristics of patients from these two independent cohorts were listed in Table 1. Most clinicopathological parameters in both cohorts were comparable, while greater proportions of patients with no smoking or cervical lymph node metastasis were included in the validation cohort as compared to those in the training counterpart.

In the training cohort, the median follow-up duration was 48.0 months (ranging from 4 to 134 month) with twenty reported deaths. In addition, 10 patients remained alive with evidence of local recurrence, cervical node metastasis or distant metastasis. In the validation cohort, the follow-up ranged from 4 to 138 months with median 45.0 months. Sixty-five patients died and 8 patients remained alive with presence of local recurrence, cervical node metastasis or distant metastasis at last follow-up. Therefore, the OS and DFS ratios were 85.5% and 62.0% (OS), 78.3% and 57.3% (DFS) for training and validation cohorts, respectively.

### Identifications of optimal cutoff values for SII, NLR and PLR from training cohort data

**Fig. 1:**
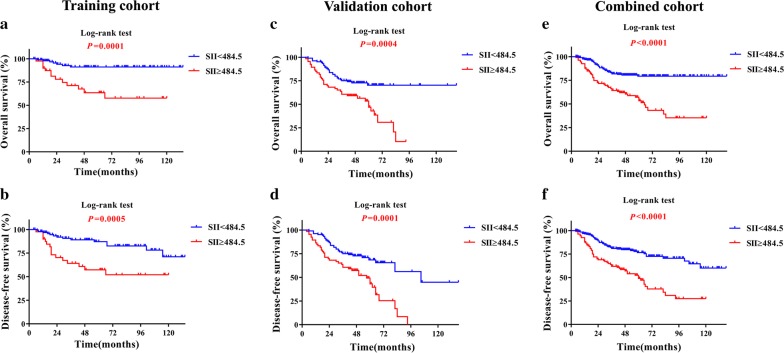
Prognostic significance of SII in patients with OSCC. The Kaplan–Meier analyses of overall survival (OS, upper panel) and disease-free survival (DFS, lower panel) in patients stratified by SII from the training (**a**, **b**), validation (**c**, **d**) and combined cohort (**e**, **f**)

The cutoff values for SII, NLR and PLR for patients stratification varied among previous studies and diverse types of cancer [9, 14, 20]. To identify optimal cutoff values for these parameters in OSCC, we utilized X-tile software to determine them by using OS as primary treatment outcome in the training cohort. As shown in Additional file 1: Fig. S1, the optimal cutoff values were 484.5 for SII, 2.9 for NLR and 170.2 for PLR respectively. Moreover, when DFS was defined as treatment outcome, these predetermined cutoff values can successfully stratify patients into high or low DFS subgroups, thus in partly substantiating their reliability and reproducibility (Fig. 1b, Additional file 2: Fig. S2B, Additional file 3: Fig. S3B). Therefore, these cutoff values were used for patient stratification in the all following analyses.

### Associations of SII, NLR and PLR with clinicopathological parameters in OSCC

Following patient stratification based on these abovementioned cutoff values, we next sought to determine the potential associations between SII, NLP, PLR and multiple clinicopathological parameters in OSCC. As listed in Table 2, high SII significantly associated with tumor size in training, validation and combined cohorts with *P* values 0.007, 0.049 and 0.002 (Chi square test) and also with advanced pathological grade in combined cohort with *P* value 0.047 (Chi square test). In addition, significant associations were detected between NLR and tumor size, gender or alcohol use in either training or validation cohort (Additional file 4: Table S1). However, no significant correlations between PLR and clinicopathological parameters were detected in both cohorts (Additional file 5: Table S2).

**Table 2:** Associations between SII and multiple clinicopathological parameters in OSCC

Variable	SII								
	Training cohort			Validation cohort			Combined cohort		
	< 484.5	≥ 484.5	*P*	< 484.5	≥ 484.5	*P*	< 484.5	≥ 484.5	*P*
No. of patients	96	42		105	66		201	108	
Age (y)									
≤ 60	36	11	0.197	38	27	0.536	74	38	0.776
> 60	60	31		67	39		127	70	
Gender									
Male	53	28	0.208	51	39	0.180	104	67	0.083
Female	43	14		54	27		97	41	
Smoking									
No	61	28	0.724	83	50	0.614	144	78	0.914
Yes	35	14		22	16		57	30	
Alcohol use									
No	70	34	0.313	90	51	0.158	160	85	0.853
Yes	26	8		15	15		41	23	
Tumor size									
T1–T2	78	25	*0.007*	88	47	*0.049*	166	72	*0.002*
T3–T4	18	17		17	19		35	36	
Pathological grade									
I	61	22	0.218	63	32	0.140	124	54	*0.047*
II–III	35	20		42	34		77	54	
Cervical nodal metastasis									
N0	78	34	0.967	73	48	0.654	151	82	0.876
N+	18	8		32	18		50	26	
Clinical stage									
I–II	65	26	0.508	65	35	0.252	130	61	0.157
III–IV	31	16		40	31		71	47	

The italic values in the table denotes *P*-values less than 0.05 with statistical significance

### Associations of SII with OS and DFS and its prognostic value for OSCC

To determine the prognostic significance of SII for OSCC, we next performed Kaplan–Meier, univariate and multivariate Cox regression analyses using data from training, validation and combined cohorts. As shown in Fig. 1a–d, when patients were stratified into groups based on SII value 484.5, patients with SII higher than 484.5 had significantly lower OS and DFS ratios as compared to those with SII less than 484.5 in both training and validation cohorts. Moreover, similar findings were obtained when data from both cohorts were combined together (Fig. 1e, f). Moreover, as indicated in Table 3, results from univariate analyses revealed that SII was significantly associated with patient overall survival in the training cohort (HR, 5.089; 95% confidence interval [CI] 2.027–12.775; *P *= 0.001), validation cohort (HR, 2.348; 95% CI 1.439–3.832; *P *= 0.001) as well as the combined cohort (HR, 3.037; 95% CI 1.973–4.673; *P *< 0.001), respectively. Consistently, similar findings were observed when DFS was used as primary treatment outcome. In addition, as expected, several well-established prognostic factors such as tumor size, pathological grade, cervical nodal metastasis and clinical stage were also individually identified as prognostic predictors for OS or DFS in OSCC patients (Table 3). To further reinforce the prognostic value of SII for OSCC and rule out other confounding factors, we performed multivariate Cox regression analyses. As indicated in Table 4 and(Additional file 6: Table S3) (in detail), our data further revealed that SII was an independent prognostic predictor for OS in the training cohort (HR, 3.887; 95% CI 1.358–11.126; *P *= 0.011), validation cohort (HR, 2.188; 95% CI 1.326–3.610; *P *= 0.002) as well as the combined cohort (HR, 2.885; 95% CI, 1.845–4.511; *P *< 0.001), respectively. Not only for OS, similar trend was also identified for DFS in the training cohort (HR, 2.814; 95% CI, 1.242–6.375; *P *= 0.013), validation cohort (HR, 2.322; 95% CI 1.444–3.732; *P *= 0.001) as well as the combined cohort (HR, 2.767; 95% CI 1.842–4.157; *P *< 0.001), respectively.

**Table 3:** Univariate survival analyses of prognostic factors associated with OS and DFS for OSCC

Variables	OS		DFS	
	HR [95% CI]	*P*	HR [95% CI]	*P*
Training cohort				
Age (> 60, ≤ 60)	0.991 (0.395–2.485)	0.985	1.223 (0.559–2.676)	0.614
Gender (male, female)	1.344 (0.536–3.369)	0.529	2.067 (0.919–4.650)	0.079
Smoking (yes, no)	0.570 (0.207–1.568)	0.276	0.983 (0.467–2.068)	0.983
Alcohol use (yes, no)	0.832 (0.278–2.488)	0.742	1.610 (0.752–3.445)	0.220
Tumor size (T3–T4, T1–T2)	3.772 (1.561–9.117)	*0.003*	2.713 (1.322–5.566)	*0.006*
Pathological grade (II–III, I)	1.691 (0.704–4.063)	0.240	1.466 (0.715–3.004)	0.296
Cervical nodal metastasis (N+, N0)	1.653 (0.600–4.550)	0.331	2.219 (1.012–4.869)	*0.047*
Clinical stage (III–IV, I–II)	2.214 (0.921–5.325)	0.076	2.634 (1.279–5.422)	*0.009*
SII (≥ 484.5, < 484.5)	5.089 (2.027–12.775)	*0.001*	3.309 (1.609–6.805)	*0.001*
NLR (≥ 2.9, < 2.9)	4.270 (1.775–10.271)	*0.001*	3.031 (1.456–6.312)	*0.003*
PLR (≥ 170.2, < 170.2)	4.493 (1.859–10.854)	*0.001*	2.397 (1.096–5.242)	*0.029*
Validation cohort				
Age (> 60, ≤ 60)	1.001 (0.607–1.651)	0.996	0.974 (0.609–1.559)	0.912
Gender (male, female)	1.124 (0.690–1.833)	0.639	1.071 (0.676–1.698)	0.769
Smoking (Yes, No)	0.779 (0.416–1.458)	0.779	0.882 (0.459–1.473)	0.510
Alcohol use (Yes, No)	1.143 (0.610–2.141)	0.677	1.100 (0.603–2.005)	0.757
Tumor size (T3–T4, T1–T2)	1.073 (0.582–1.976)	0.821	1.084 (0.602–1.954)	0.787
Pathological grade (II–III, I)	2.039 (1.247–3.334)	*0.005*	1.789 (1.125–2.844)	*0.014*
Cervical nodal metastasis (N+, N0)	1.137 (0.663–1.949)	0.641	1.116 (0.662–1.883)	0.680
Clinical stage (III–IV, I–II)	1.209 (0.733–1.994)	0.457	1.148 (0.707–1.864)	0.576
SII (≥ 484.5, < 484.5)	2.348 (1.439–3.832)	*0.001*	2.431 (1.525–3.876)	< *0.001*
NLR (≥ 2.9, < 2.9)	2.101 (1.263–3.496)	*0.004*	2.072 (1.275–3.369)	*0.003*
PLR (≥ 170.2, < 170.2)	2.633 (1.610–4.307)	< *0.001*	2.423 (1.508–3.893)	< *0.001*
Combined cohort				
Age (> 60, ≤ 60)	0.971 (0.626–1.507)	0.897	0.998 (0.669–1.490)	0.992
Gender (male, female)	1.090 (0.710–1.674)	0.693	1.192 (0.805–1.764)	0.380
Smoking (yes, no)	0.598 (0.351–1.018)	0.058	0.750 (0.477–1.177)	0.210
Alcohol use (yes, no)	0.942 (0.547–1.623)	0.830	1.143 (0.718–1.819)	0.573
Tumor size (T3–T4, T1–T2)	1.439 (0.897–2.309)	0.131	1.387 (0.898–2.142)	0.140
Pathological grade (II–III, I)	1.913 (1.248–2.933)	*0.003*	1.609 (1.093–2.369)	*0.016*
Cervical nodal metastasis (N + , N0)	1.337 (0.833–2.145)	0.229	1.410 (0.915–2.172)	0.119
Clinical stage (III–IV, I–II)	1.437 (0.935–2.208)	0.098	1.462 (0.987–2.166)	0.058
SII (≥ 484.5, < 484.5)	3.037 (1.973–4.673)	< *0.001*	2.801 (1.896–4.138)	< *0.001*
NLR (≥ 2.9, < 2.9)	2.587 (1.672–4.005)	< *0.001*	2.378 (1.588–3.562)	< *0.001*
PLR (≥ 170.2, < 170.2)	3.250 (2.116–4.993)	< *0.001*	2.545 (1.703–3.803)	< *0.001*

*HR* hazard ratio, *CI* confidence interval

**Table 4:** Multivariate survival analyses of prognostic factors associated with OS and DFS for OSCC

Variables	OS		DFS	
	HR [95% CI]ª	*P*ª	HR [95% CI]ª	*P*ª
Training cohort				
SII (≥ 484.5, < 484.5)	3.887 (1.358–11.126)	0.011	2.814 (1.242–6.375)	0.013
NLR (≥ 2.9, < 2.9)	3.114 (1.148–8.446)	0.026	2.328 (1.000–5.422)	0.050
PLR (≥ 170.2, < 170.2)	5.678 (2.130–15.135)	0.001	3.318 (1.414–7.788)	0.006
Validation cohort				
SII (≥ 484.5, < 484.5)	2.188 (1.326–3.610)	0.002	2.322 (1.444–3.732)	0.001
NLR (≥ 2.9, < 2.9)	1.982 (1.150–3.416)	0.014	2.015 (1.204–3.373)	0.008
PLR (≥ 170.2, < 170.2)	2.697 (1.628–4.466)	< 0.001	2.496 (1.538–4.050)	< 0.001
Combined cohort				
SII (≥ 484.5, < 484.5)	2.885 (1.845–4.511)	< 0.001	2.767 (1.842–4.157)	< 0.001
NLR (≥ 2.9, < 2.9)	2.458 (1.547–3.903)	< 0.001	2.305 (1.499–3.543)	< 0.001
PLR (≥ 170.2, < 170.2)	3.203 (2.071–4.954)	< 0.001	2.600 (1.727–3.912)	< 0.001

*HR* hazard ratio, *CI* confidence interval

ªAdjusted for age, gender, smoking, alcohol use, tumor size, pathological grade, cervical nodal metastasis, clinical stage in logistic regression models

### Associations of NLR and PLR with OS and DFS and their prognostic values for OSCC

In addition to SII, we next proceeded to determine the associations of NLR and PLR with patient survival and their prognostic values for OSCC. As shown in Additional file 2: Fig. S2, patients with high NLR had significantly reduced OS and DFS compared to those with low NLR in training, validation and combined cohorts (Kaplan–Meier analyses, *P *< 0.01). Moreover, patients with high PLR had significant lower OS and DFS relative those with low PLR in training, validation and combined cohorts (Kaplan–Meier analyses, *P *< 0.05, Additional file 3: Fig. S3). To further substantiate the prognostic significance of NLR and PLR we performed both univariate and multivariate analyses and revealed that both NLR and PLR were independent prognostic predictors for OS and DFS in patients with OSCC (Tables 3 and 4, Additional file 7: Table S4 and Additional file 8: Table S5).

### Predictive abilities of SII, NLR and PLR for OSCC prognosis

**Fig. 2:**
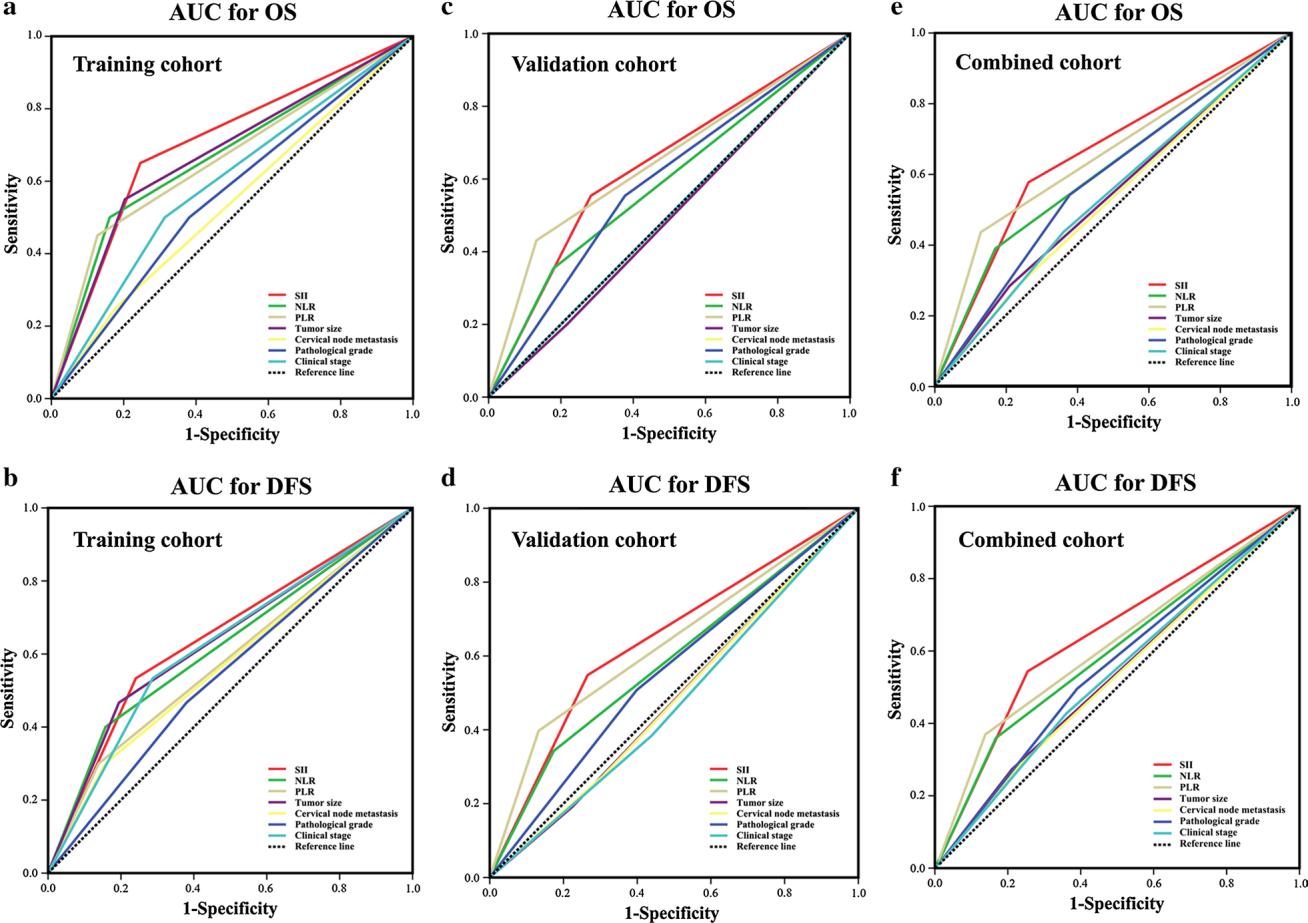
Predictive ability of SII for prognosis of patients with OSCC. The sensitivity and specificity of SII and other clinicopathological parameters in prognostic prediction (OS, the upper panel; DFS, the lower panel) were estimated by ROC curves in training (**a**, **b**), validation (**c**, **d**) and combined cohort (**e**, **f**)

Having demonstrated the prognostic values of SII, NLR as well as PLR for OSCC, we next sought to compare the discrimination abilities of these three prognostic parameters with four well-established prognostic factors including tumor size, cervical nodal metastasis, pathological grade and clinical stage by the AUC for OS and DFS. As shown in Fig. 2E and Additional file 9: Fig. S4E, when predicted OS for patients in combined cohort, the AUC for SII, NLR and PLR were 0.657, 0.609, 0.653, while the AUC for other four parameters were less than 0.6. Furthermore, when predicted DFS for patients in combined cohort (Fig. 2f, Additional file 9: Fig. S4F), the AUC for SII, NLR and PLR were 0.646, 0.597, 0.617, while the AUC for other four parameters were less than 0.56. Similar findings were also observed in training or validation cohort (Fig. 2a–d, Additional file 9: Fig. S4A–D). Of particular interest, SII remains the superior prognostic predictor with the highest sensitivity and specificity for OS and DFS. Collectively, these findings suggested that, in terms of predictive ability for prognosis, SII, NLR and PLR were superior or comparable to those previously well-established prognostic factors.

## Discussion

The dismal long-term survival and paucity of optimized biomarkers for OSCC highlight the pressing need to identify effective prognostic biomarkers and regime to better patient stratification and treatment planning [1, 2]. Here, we determined the prognostic values of systemic inflammation-immune biomarkers SII, NLR and PLR in two independent cohorts of patients with resectable OSCC and found that these inflammation and immune-related scores significantly associated with overall and disease-free survival and also served as independent prognostic predictors with satisfactory sensitivity and specificity for OSCC. Our finding provides further support for the prognostic significance of systemic inflammation-immune biomarkers in OSCC.

Accumulating evidence has revealed the prognostic values of SII, NLR, PLR and other inflammation-related parameters in several types of cancer [9, 14, 21]. Previous studies have indicated that high SII, NLR and PLR significantly associated with adverse survival in hepatocellular carcinoma, esophageal squamous cell carcinoma, pancreatic cancer, melanoma and small cell lung cancer [16, 17, 20, 24, 25]. Eltohami YI, et al. reported systemic inflammation score (SIS) based on serum albumin levels and the lymphocyte-to-monocyte ratio and found that higher SIS significantly associated with poor prognosis in OSCC [26]. However, the cutoff values for these prognostic predictors for patient stratification varied among diverse studies and their optimal values for each cancer type might be a prerequisite before they are translated into clinical practice [9, 14, 21]. Here, the optimal cutoff value for SII (484.5) in the training cohort using X-tile software was firstly identified, which was generally similar with its values in other types of solid cancer such as 330 in hepatocellular carcinoma [16], 403 in nasopharyngeal carcinoma [27] and 410 in esophageal squamous cell carcinoma [28]. This cutoff value was subsequently utilized for patient stratification and also verified in another independent validation or combined cohort. We believe that cutoff value for SII might be variable and cancer-specific, given the different genetic background, etiological factors and cancer cell of origin. In support to this idea, previous meta-regression analyses have revealed that this variation regarding SII cutoff value might be not in association with reported HR for survival [14]. Of course, although the cutoff value for SII identified here was reasonable, it might be further optimized or validated in a large population of patients. Consistent with previous findings regarding prognostic significance of SII, our data reveal that high SII positively associates with reduced overall and disease-free survival in OSCC. Cox-regression analyses further identify SII as an important independent prognostic factor for OSCC. Furthermore, as compared to other previously established prognostic biomarkers, SII might be superior or comparable in predicting clinical outcomes. In addition to SII, other inflammatory and immune-related parameter such as NLR and PLR have been reported with prognostic significance among diverse cancers [9, 14]. Previously, high preoperative NLR and PLR significantly associated with increased risk of death and worse survival in primary OSCC [29]. In line with these results, our findings further reveal that NLR and PLR, these two ratios significantly associated with unfavorable outcome as evidenced by reduced OS and DFS. Moreover, they are identified as independent prognostic factors for patient survival, thus also supporting their prognostic values for OSCC. Notably, the cutoff value for NLR in our study was 2.9 which was lower as compared to some other studies such as 5 in oropharyngeal and non-oropharyngeal HNSCC [30]. We reasoned that inherent heterogeneity of HNSCC and OSCC, diverse genetic background as well as underlying etiological factors might be responsible for this difference. However, the sensitivity and specificity of prognostic prediction for SII were superior or comparable to NLR and PLR, presumably due to its integrated evaluation on all three types of cells. Together, these findings indicate that these systemic immune-inflammation parameters like SII can serve as previously unappreciated prognostic biomarkers for OSCC with promising translational significance.

Considering SII, NLR and PLR as integrated indicator based on peripheral neutrophils, lymphocytes and platelets, their prognostic predictive value for cancer might be elucidated by the functional status of these three types of cells in patients [16, 21]. High SII might result from neutrophilia, thrombocythemia and lymphopenia, likely reflecting a combination of elevated non-specific inflammation and dysfunctional immune system. Thrombocythemia and lymphopenia usually promote cancer progression and associate with unfavorable survival [31–33]. In line with these notions, it has been increasingly appreciated that deregulated inflammation or immune deficit drives tumor growth, invasion and metastasis as well as therapeutic resistance in cancer [7, 8]. For example, inflammatory cells were activated by increased TNF-α in a paracrine manner, which tipped balance toward cancer invasion leading to decreased survival in OSCC [34]. Indeed, neutrophils, lymphocytes and platelets are critically involved in cancer progression not only in local tumor microenvironment but also in peripheral systemic bloodstream. Neutrophils can promote cancer cell proliferation, invasion, immune evasion, and angiogenesis by secreting growth factors, while platelets directly interacts with cancer cells, synergistically triggers TGF-β, NF-kB pathways in cancer and facilitates EMT and metastasis [35–37]. Moreover, lymphocytes as fundamental immune cells engage in cell-mediated immunologic destruction of cancer cells, although diverse subtypes of lymphocytes differ in their accurate roles against cancer [38, 39]. Therefore, reduced lymphocytes is indicative of impaired immune surveillance and favorable tumor environment for tumor dissemination.

Previous reports have shown that SII, NLR and PLR are significantly correlated with aggressive features of cancer [16, 17]. For example, patients with high SII were more likely have larger tumor size, poor differentiation, tumor recurrence/metastasis and advanced stage in hepatocellular carcinoma, pancreatic cancer and germ-cell tumor [16–18]. In agreement with these findings, our results revealed that SII positively correlated with large tumor size and high pathological grade, while NLR positively correlated with large tumor size in OSCC. However, no significant correlations were detected between PLR and clinicopathological features. It’s conceivable that these neutrophils, lymphocytes and platelets execute their diverse functions both locally and systemically, ultimately fuelling cancer initiation and progression [37, 38].

However, our study has certain limitations including its retrospective nature and relatively small number of patients. Bias for patient selection and group difference between training and validation cohort inevitably remained. Complete clinicopathological information, long-term follow-up data and training-validation cohort design might in part compensate this weakness. Further prospective studies in larger samples are warranted to verify the prognostic value of SII for OSCC. In addition, some critical prognostic factors such as status of surgical margin, depth of invasion, presence of extracapsular extension or perineural invasion which were obtained after surgical resection of primary lesions were not included here for univariate and multivariate survival analyses. Moreover, SII is a dynamic score which may change during cancer progression and therapeutic intervention [40]. Monitoring SII during treatment course may provide more interesting information about the status of inflammatory and immune response of host as well as clinical response to therapy [40, 41]. Thus, it remains as an open and interesting question to incorporate SII into the prognostic regime to assess patient outcome, risk of disease progression as well as therapeutic response in the clinical settings. Furthermore, we speculate that evaluation of pre-treatment SII might offer beneficial information in treatment planning and guiding in patients with OSCC, although current data concerning the prognostic predictive values of SII in OSCC patients underwent diverse treatment approaches like surgery alone or combination treatment with surgery and chemotherapy/radiotherapy are still lacking.

## Conclusions

In conclusion, our results confirmed that SII qualifies as a novel independent prognostic predictor of survival for patients with resectable OSCC. Due to its superior attributes of simplicity, reproducibility, easy calculation and low cost, SII holds great potentials as a robust and promising parameter for assessing OSCC prognosis. Further larger and properly designed prospective study is warranted to validate the prognostic value of SII in OSCC before its entrance into daily clinical practice.

## Additional files

**Additional file 1: Fig. S1.** The optimal cutoff values of SII (**A**), NLR (**B**) and PLR (**C**) were determined by X-tile software (Yale University, New Haven, CT) using OS as the primary outcome in patients from the training cohort.

**Additional file 2: Fig. S2.** Prognostic significance of NLR in patients with OSCC. The Kaplan–Meier analyses of overall survival (OS, upper panel)and disease-free survival (DFS, lower panel) in patients stratified by NLR from the training (**A**, **B**), validation (**C**, **D**) and combined cohort (**E**, **F**).

**Additional file 3: Fig. S3.** Prognostic significance of PLR in patients with OSCC. The Kaplan–Meier analyses of overall survival (OS, upper panel)and disease-free survival (DFS, lower panel) in patients stratified by NLR from the training (**A**, **B**), validation (**C**, **D**) and combined cohort (**E**, **F**).

**Additional file 4: Table S1.** Associations between NLR and multiple clinicopathological parameters in OSCC.

**Additional file 5: Table S2.** Associations between PLR and multiple clinicopathological parameters in OSCC.

**Additional file 6: Table S3.** Multivariate survival analyses of prognostic factors associated with OS and DFS for OSCC.

**Additional file 7: Table S4.** Multivariate survival analyses of prognostic factors associated with OS and DFS for OSCC.

**Additional file 8: Table S5.** Multivariate survival analyses of prognostic factors associated with OS and DFS for OSCC.

**Additional file 9: Fig. S4.** AUC values with 95% CI for each parameter in prognostic prediction of OS (upper panel) and DFS (lower panel) in patients from training (**A**, **B**), validation (**C**, **D**) and combined cohort (**E**, **F**). *Indicates *P*<0.05 as compared to SII.

## Abbreviations

CBP: C-reactive protein
DFS: disease-free survival
NLR: neutrophil–lymphocyte ratio
OSCC: oral squamous cell carcinoma
OS: overall survival
PLR: platelet–lymphocyte ratio
SII: systemic immune-inflammation index
