# Supplementary figures and images for: Preoperative systemic immune-inflammation index predicts prognosis of patients with oral squamous cell carcinoma after curative resection
Source: J Transl Med. 2018 Dec 18;16:365. doi: 10.1186/s12967-018-1742-x (PMC6299596; doi:10.1186/s12967-018-1742-x)

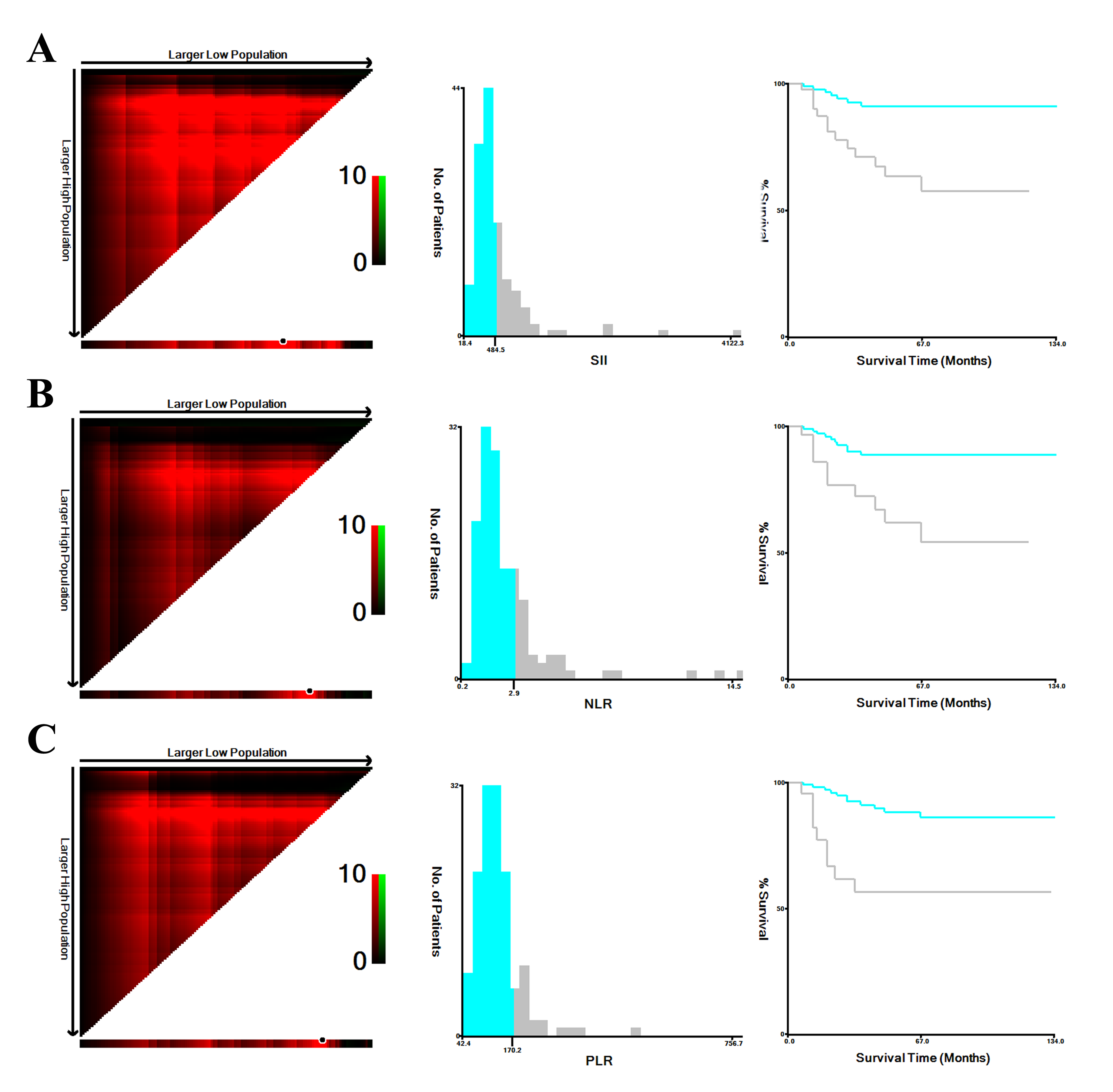

Supplement: Supplementary file 1 — Additional file 1: Fig. S1. The optimal cutoff values of SII (A), NLR (B) and PLR (C) were determined by X-tile software (Yale University, New Haven, CT) using OS as the primary outcome in patients from the training cohort. [file 12967_2018_1742_MOESM1_ESM.tif]

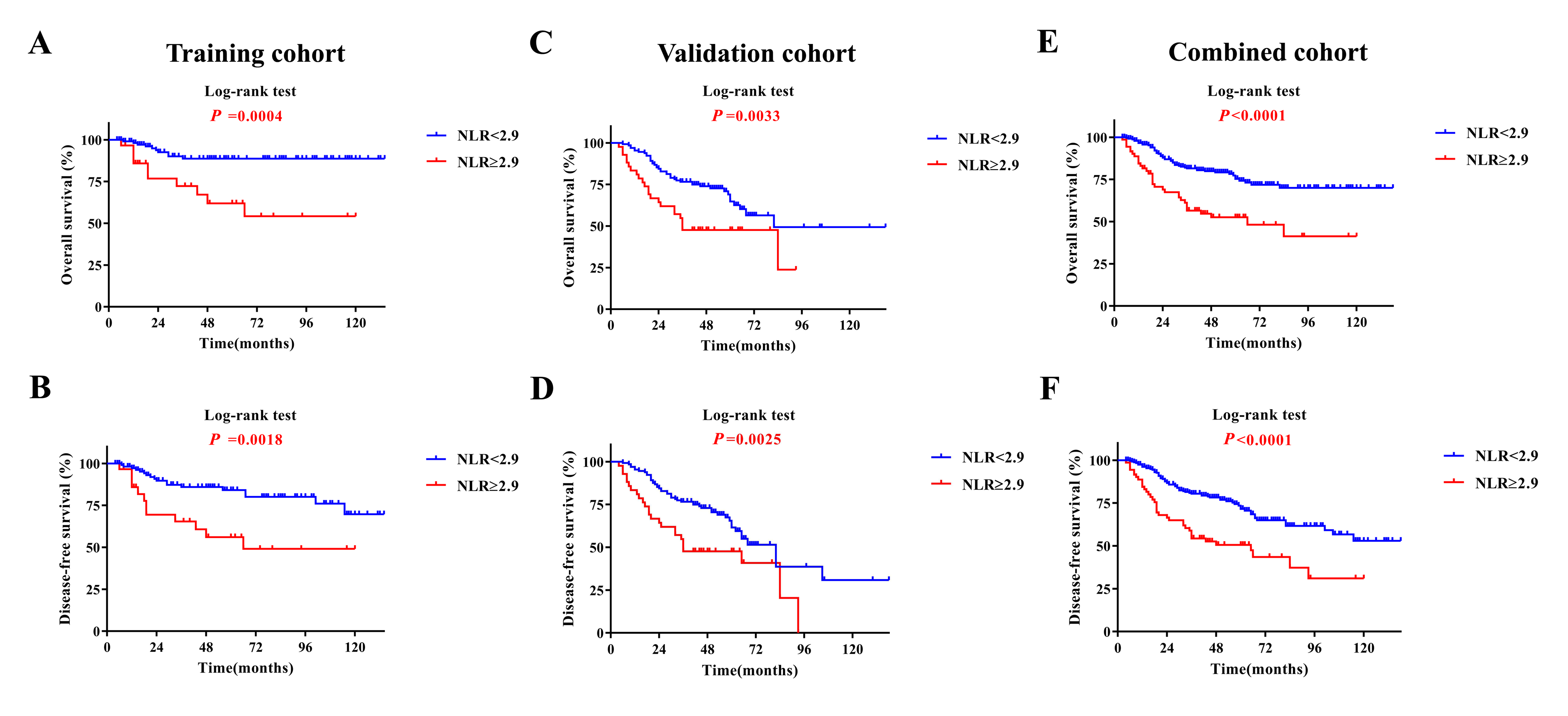

Supplement: Supplementary file 2 — Additional file 2: Fig. S2. Prognostic significance of NLR in patients with OSCC. The Kaplan–Meier analyses of overall survival (OS, upper panel)and disease-free survival (DFS, lower panel) in patients stratified by NLR from the training (A, B), validation (C, D) and combined cohort (E, F). [file 12967_2018_1742_MOESM2_ESM.tif]

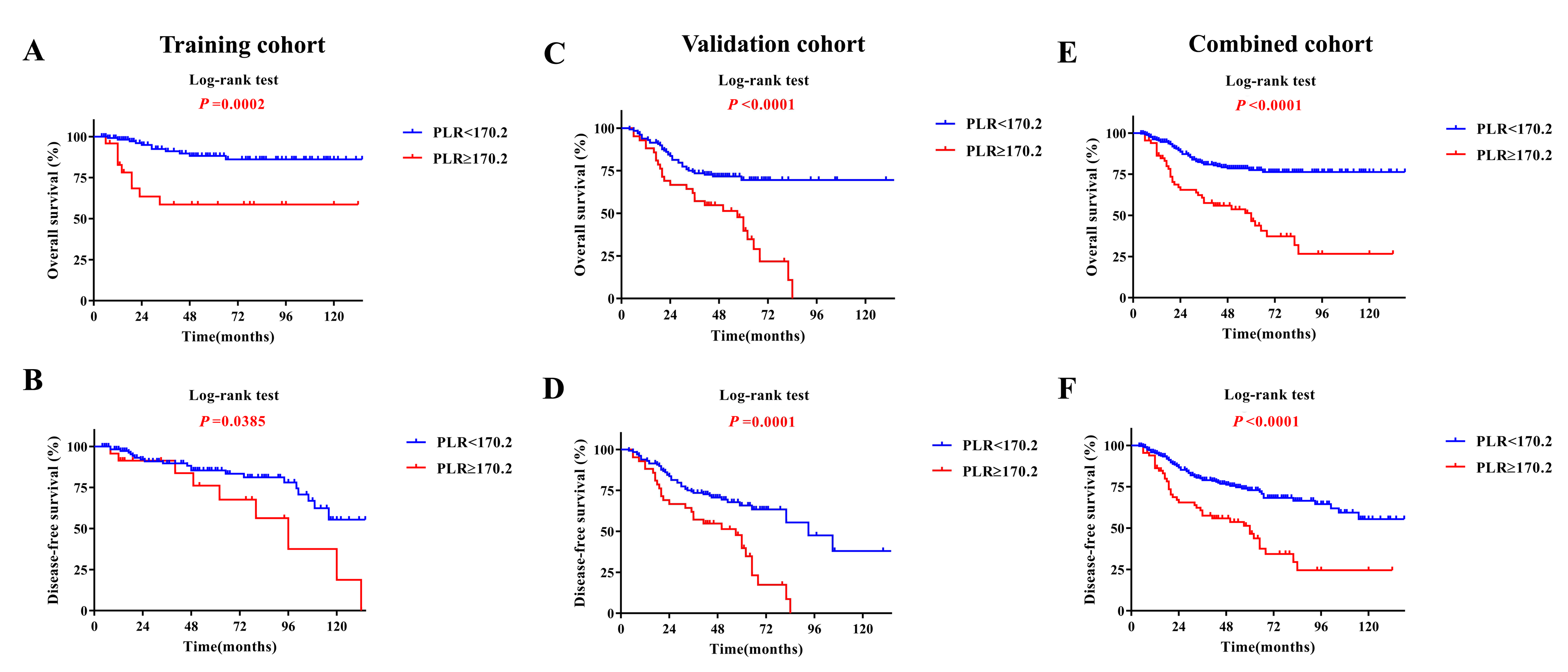

Supplement: Supplementary file 3 — Additional file 3: Fig. S3. Prognostic significance of PLR in patients with OSCC. The Kaplan–Meier analyses of overall survival (OS, upper panel)and disease-free survival (DFS, lower panel) in patients stratified by NLR from the training (A, B), validation (C, D) and combined cohort (E, F). [file 12967_2018_1742_MOESM3_ESM.tif]

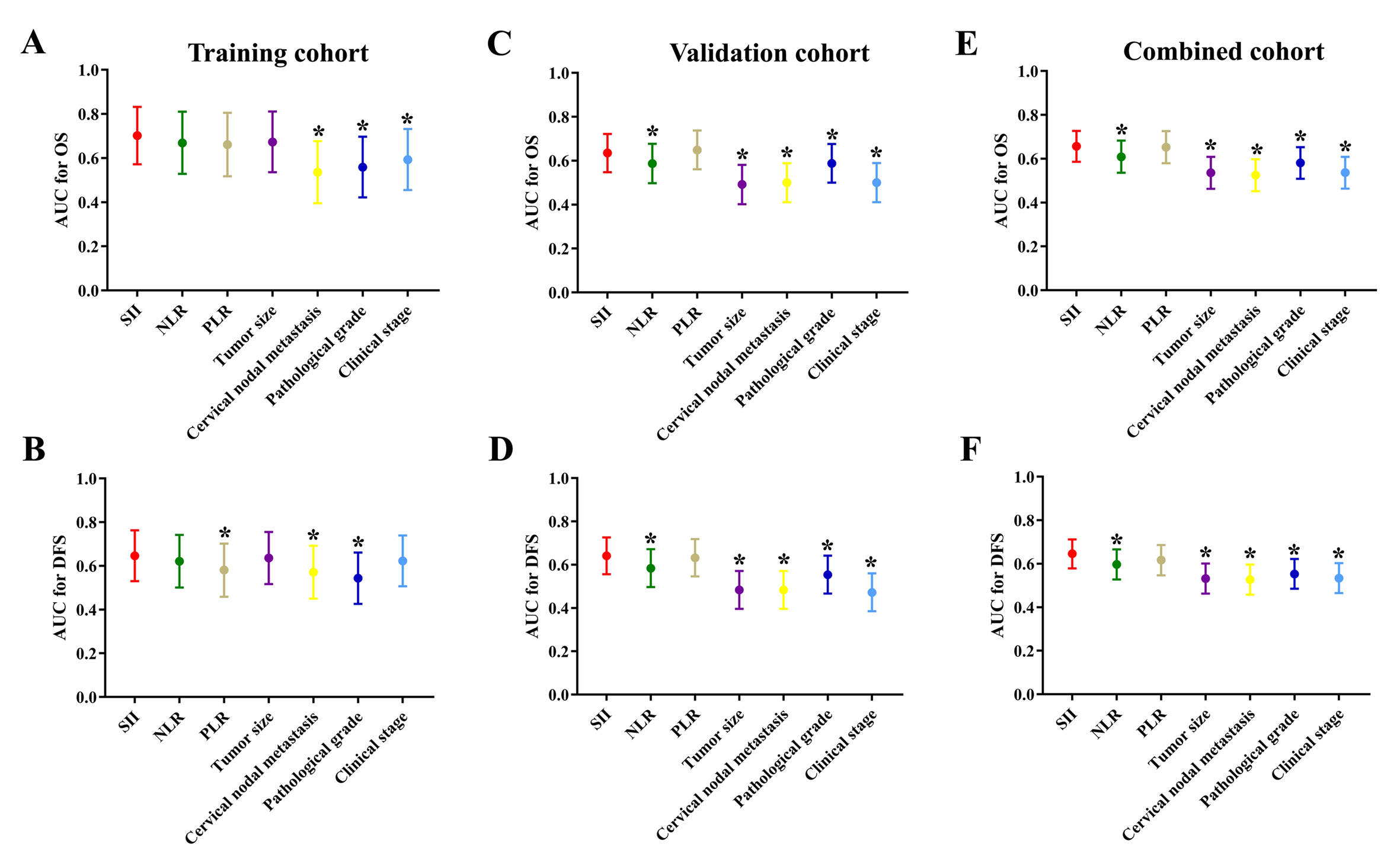

Supplement: Supplementary file 9 — Additional file 9: Fig. S4. AUC values with 95% CI for each parameter in prognostic prediction of OS (upper panel) and DFS (lower panel) in patients from training (A, B), validation (C, D) and combined cohort (E, F). *Indicates P<0.05 as compared to SII. [file 12967_2018_1742_MOESM9_ESM.tif]
