# Supplementary material for: Preoperative systemic immune-inflammation index predicts prognosis of patients with oral squamous cell carcinoma after curative resection
Source: J Transl Med. 2018 Dec 18;16:365. doi: 10.1186/s12967-018-1742-x (PMC6299596; doi:10.1186/s12967-018-1742-x)
Supplement: Supplementary file 4 — Additional file 4: Table S1. Associations between NLR and multiple clinicopathological parameters in OSCC. [file 12967_2018_1742_MOESM4_ESM.docx]

| **Additional Table S1. Associations between NLR and multiple clinicopathological parameters in OSCC** | | | | | | | | | | | | | | |
| --- | --- | --- | --- | --- | --- | --- | --- | --- | --- | --- | --- | --- | --- | --- |
| **Variable** | |  | **NLR** | | | | | | | | | | | |
|  |  |  | **Training cohort** | | |  | **Validation cohort** | | |  | **Combined cohort** | | |  |
|  |  |  | **<2.9** | **≥2.9** | ***P*** |  | **<2.9** | **≥2.9** | ***P*** |  | **<2.9** | **≥2.9** | ***P*** |  |
| **No. of patients** | |  | 109 | 29 |  |  | 129 | 42 |  |  | 238 | 71 |  |  |
| **Age (y)** | ≤60 |  | 38 | 9 | 0.699 |  | 48 | 17 | 0.705 |  | 86 | 26 | 0.941 |  |
|  | >60 |  | 71 | 20 |  |  | 81 | 25 |  |  | 152 | 45 |  |  |
| **Gender** | Male |  | 59 | 22 | **0.035** |  | 65 | 25 | 0.303 |  | 124 | 47 | **0.036** |  |
|  | Female |  | 50 | 7 |  |  | 64 | 17 |  |  | 114 | 24 |  |  |
| **Smoking** | No |  | 71 | 18 | 0.759 |  | 100 | 33 | 0.887 |  | 171 | 51 | 0.998 |  |
|  | Yes |  | 38 | 11 |  |  | 29 | 9 |  |  | 67 | 20 |  |  |
| **Alcohol use** | No |  | 83 | 21 | 0.678 |  | 111 | 30 | **0.031** |  | 194 | 51 | 0.077 |  |
|  | Yes |  | 26 | 8 |  |  | 18 | 12 |  |  | 44 | 20 |  |  |
| **Tumor size** | T1-T2 |  | 87 | 16 | **0.007** |  | 108 | 27 | **0.007** |  | 195 | 43 | **0.002** |  |
|  | T3-T4 |  | 22 | 13 |  |  | 21 | 15 |  |  | 43 | 28 |  |  |
| **Pathological grade** | I |  | 67 | 16 | 0.538 |  | 76 | 19 | 0.121 |  | 143 | 35 | 0.106 |  |
|  | II-III |  | 42 | 13 |  |  | 53 | 23 |  |  | 95 | 36 |  |  |
| **Cervical nodal**  **metastasis** | N0 |  | 90 | 22 | 0.412 |  | 88 | 33 | 0.200 |  | 178 | 55 | 0.646 |  |
|  | N+ |  | 19 | 7 |  |  | 41 | 9 |  |  | 60 | 16 |  |  |
| **Clinical stage** | I-II |  | 74 | 17 | 0.349 |  | 78 | 22 | 0.356 |  | 152 | 39 | 0.174 |  |
|  | III-IV |  | 35 | 12 |  |  | 51 | 20 |  |  | 86 | 32 |  |  |
