# Supplementary material for: Preoperative systemic immune-inflammation index predicts prognosis of patients with oral squamous cell carcinoma after curative resection
Source: J Transl Med. 2018 Dec 18;16:365. doi: 10.1186/s12967-018-1742-x (PMC6299596; doi:10.1186/s12967-018-1742-x)
Supplement: Supplementary file 5 — Additional file 5: Table S2. Associations between PLR and multiple clinicopathological parameters in OSCC. [file 12967_2018_1742_MOESM5_ESM.docx]

| **Variable** | | **PLR** | | | | | | | | | | | |
| --- | --- | --- | --- | --- | --- | --- | --- | --- | --- | --- | --- | --- | --- |
|  |  | **Training cohort** | | |  | **Validation cohort** | | |  | **Combined cohort** | | |  |
|  |  | **<170.2** | **≥170.2** | ***P*** |  | **<170.2** | **≥170.2** | ***P*** |  | **<170.2** | **≥170.2** | ***P*** |  |
| **No. of patients** | | 114 | 24 |  |  | 129 | 42 |  |  | 243 | 66 |  |  |
| **Age (y)** | ≤60 | 38 | 9 | 0.695 |  | 50 | 15 | 0.724 |  | 88 | 24 | 0.982 |  |
|  | >60 | 76 | 15 |  |  | 79 | 27 |  |  | 155 | 42 |  |  |
| **Gender** | Male | 67 | 14 | 0.968 |  | 66 | 24 | 0.500 |  | 133 | 38 | 0.680 |  |
|  | Female | 47 | 10 |  |  | 63 | 18 |  |  | 110 | 28 |  |  |
| **Smoking** | No | 72 | 17 | 0.475 |  | 100 | 33 | 0.887 |  | 172 | 50 | 0.425 |  |
|  | Yes | 42 | 7 |  |  | 29 | 9 |  |  | 71 | 16 |  |  |
| **Alcohol use** | No | 83 | 21 | 0.192* |  | 106 | 35 | 0.863 |  | 189 | 56 | 0.209 |  |
|  | Yes | 31 | 3 |  |  | 23 | 7 |  |  | 54 | 10 |  |  |
| **Tumor size** | T1-T2 | 88 | 15 | 0.133 |  | 105 | 30 | 0.169 |  | 193 | 45 | 0.054 |  |
|  | T3-T4 | 26 | 9 |  |  | 24 | 12 |  |  | 50 | 21 |  |  |
| **Pathological grade** | I | 70 | 13 | 0.510 |  | 71 | 24 | 0.812 |  | 141 | 37 | 0.775 |  |
|  | II-III | 44 | 11 |  |  | 58 | 18 |  |  | 102 | 29 |  |  |
| **Cervical nodal**  **metastasis** | N0 | 91 | 21 | 0.567* |  | 89 | 32 | 0373 |  | 180 | 53 | 0.297 |  |
|  | N+ | 23 | 3 |  |  | 40 | 10 |  |  | 63 | 13 |  |  |
| **Clinical stage** | I-II | 75 | 16 | 0.934 |  | 76 | 24 | 0.840 |  | 151 | 40 | 0.820 |  |
|  | III-IV | 39 | 8 |  |  | 53 | 18 |  |  | 92 | 26 |  |  |

**Additional Table S2. Associations between PLR and multiple clinicopathological parameters in OSCC**

* Fisher exact test
