# Supplementary material for: Preoperative systemic immune-inflammation index predicts prognosis of patients with oral squamous cell carcinoma after curative resection
Source: J Transl Med. 2018 Dec 18;16:365. doi: 10.1186/s12967-018-1742-x (PMC6299596; doi:10.1186/s12967-018-1742-x)
Supplement: Supplementary file 6 — Additional file 6: Table S3. Multivariate survival analyses of prognostic factors associated with OS and DFS for OSCC. [file 12967_2018_1742_MOESM6_ESM.docx]

| **Additional Table S3. Multivariate survival analyses of prognostic factors associated with OS and DFS for OSCC** | | | | | | | | | | | |
| --- | --- | --- | --- | --- | --- | --- | --- | --- | --- | --- | --- |
| **Variables** | | **OS** | | | |  | | **DFS** | | | |
|  |  | **HR [95% CI]** | | ***P*** | |  | | **HR [95% CI]** | | ***P*** | |
| **Training cohort** | |  | |  | |  | |  | |  | |
| Age (>60, ≤60) | | 1.101(0.399-3.036) | | 0.853 | |  | | 1.361(0.585-3.167) | | 0.474 | |
| Gender (male, female) | | 1.236(0.440-3.472) | | 0.687 | |  | | 1.747(0.679-4.492) | | 0.247 | |
| Smoking (Yes, No) | | 0.312(0.069-1.410) | | 0.130 | |  | | 0.374(0.120-1.170) | | 0.091 | |
| Alcohol use (Yes, No) | | 1.851(0.354-9.692) | | 0.466 | |  | | 2.455(0.764-7.895) | | 0.132 | |
| Tumor size (T3-T4, T1-T2) | | 2.630(0.707-9.775) | | 0.149 | |  | | 1.461(0.503-4.241) | | 0.486 | |
| Pathological grade (II-III, I) | | 1.036(0.393-2.731) | | 0.942 | |  | | 1.073(0.489-2.353) | | 0.861 | |
| Cervical nodal metastasis (N+, N0) | | 1.675(0.443-6.327) | | 0.447 | |  | | 2.017(0.655-6.206) | | 0.221 | |
| Clinical stage (III-IV, I-II) | | 0.805(0.188-3.448) | | 0.770 | |  | | 1.304(0.381-4.454) | | 0.672 | |
| SII (≥484.5, <484.5) | | 3.887(1.358-11.126) | | **0.011** | |  | | 2.814(1.242-6.375) | | **0.013** | |
| **Validation cohort** | |  | |  | |  | |  | |  | |
| Age (>60, ≤60) | | 0.937(0.553-1.588) | | 0.810 | |  | | 0.917(0.559-1.503) | | 0.731 | |
| Gender (male, female) | | 1.113(0.623-1.988) | | 0.718 | |  | | 1.063(0.611-1.849) | | 0.830 | |
| Smoking (Yes, No) | | 0.529(0.219-1.277) | | 0.157 | |  | | 0.619(0.268-1.431) | | 0.262 | |
| Alcohol use (Yes, No) | | 1.680(0.687-4.110) | | 0.256 | |  | | 1.485(0.628-3.513) | | 0.368 | |
| Tumor size (T3-T4, T1-T2) | | 0.643(0.231-1.789) | | 0.398 | |  | | 0.799(0.307-2.078) | | 0.645 | |
| Pathological grade (II-III, I) | | 1.969(1.162-3.335) | | **0.012** | |  | | 1.708(1.037-2.814) | | **0.036** | |
| Cervical nodal metastasis (N+, N0) | | 0.819(0.270-2.482) | | 0.724 | |  | | 1.016(0.357-2.893) | | 0.976 | |
| Clinical stage (III-IV, I-II) | | 1.345(0.389-4.650) | | 0.640 | |  | | 1.049(0.322-3.415) | | 0.936 | |
| SII (≥484.5, <484.5) | | 2.188(1.326-3.610) | | **0.002** | |  | | 2.322(1.444-3.732) | | **0.001** | |
| **Combined cohort** | |  | |  | |  | |  | |  | |
| Age (>60, ≤60) | | 0.964(0.613-1.518) | | 0.875 | |  | | 1.007(0.668-1.518) | | 0.975 | |
| Gender (male, female) | | 1.132(0.693-1.848) | | 0.620 | |  | | 1.156(0.733-1.823) | | 0.533 | |
| Smoking (Yes, No) | | 0.396(0.193-0.814) | | **0.012** | |  | | 0.474(0.255-0.880) | | **0.018** | |
| Alcohol use (Yes, No) | | 1.673(0.795-3.521) | | 0.175 | |  | | 1.792(0.950-3.383) | | 0.072 | |
| Tumor size (T3-T4, T1-T2) | | 0.913(0.437-1.911) | | 0.810 | |  | | 0.886(0.455-1.725) | | 0.722 | |
| Pathological grade (II-III, I) | | 1.599(1.018-2.510) | | **0.041** | |  | | 1.340(0.891-2.017) | | 0.160 | |
| Cervical nodal metastasis (N+, N0) | | 1.185(0.526-2.669) | | 0.682 | |  | | 1.281(0.607-2.702) | | 0.516 | |
| Clinical stage (III-IV, I-II) | | 1.116(0.445-2.797) | | 0.816 | |  | | 1.148(0.501-2.632) | | 0.744 | |
| SII (≥484.5, <484.5) | | 2.885(1.845-4.511) | | **<0.001** | |  | | 2.767(1.842-4.157) | | **<0.001** | |
| HR, hazard ratio; CI, confidence interval. | |  | |  | |  | |  | |  | |
