# Supplementary material for: Preoperative systemic immune-inflammation index predicts prognosis of patients with oral squamous cell carcinoma after curative resection
Source: J Transl Med. 2018 Dec 18;16:365. doi: 10.1186/s12967-018-1742-x (PMC6299596; doi:10.1186/s12967-018-1742-x)
Supplement: Supplementary file 7 — Additional file 7: Table S4. Multivariate survival analyses of prognostic factors associated with OS and DFS for OSCC. [file 12967_2018_1742_MOESM7_ESM.docx]

| **Additional Table S4. Multivariate survival analyses of prognostic factors associated with OS and DFS for OSCC** | | | | | | | | | | | |
| --- | --- | --- | --- | --- | --- | --- | --- | --- | --- | --- | --- |
| **Variables** | | **OS** | | | |  | | **DFS** | | | |
|  |  | **HR [95% CI]** | | ***P*** | |  | | **HR [95% CI]** | | ***P*** | |
| **Training cohort** | |  | |  | |  | |  | |  | |
| Age (>60, ≤60) | | 1.259(0.458-3.460) | | 0.655 | |  | | 1.626(0.696-3.799) | | 0.261 | |
| Gender (male, female) | | 1.246(0.436-3.558) | | 0.681 | |  | | 1.891(0.733-4.880) | | 0.188 | |
| Smoking (Yes, No) | | 0.313(0.067-1.465) | | 0.140 | |  | | 0.386(0.124-1.203) | | 0.101 | |
| Alcohol use (Yes, No) | | 1.350(0.265-6.893) | | 0.718 | |  | | 1.863(0.601-5.777) | | 0.281 | |
| Tumor size (T3-T4, T1-T2) | | 3.346(0.780-14.353) | | 0.104 | |  | | 1.473(0.471-4.611) | | 0.505 | |
| Pathological grade (II-III, I) | | 1.195(0.468-3.050) | | 0.709 | |  | | 1.204(0.561-2.584) | | 0.634 | |
| Cervical nodal metastasis (N+, N0) | | 1.274(0.328-4.944) | | 0.727 | |  | | 1.621(0.508-5.178) | | 0.415 | |
| Clinical stage (III-IV, I-II) | | 0.780(0.155-3.924) | | 0.763 | |  | | 1.578(0.420-5.939) | | 0.500 | |
| NLR (≥2.9, <2.9) | | 3.114(1.148-8.446) | | **0.026** | |  | | 2.328(1.000-5.422) | | **0.050** | |
| **Validation cohort** | |  | |  | |  | |  | |  | |
| Age (>60, ≤60) | | 0.910(0.535-1.550) | | 0.729 | |  | | 0.887(0.539-1.462) | | 0.639 | |
| Gender (male, female) | | 1.202(0.674-2.144) | | 0.534 | |  | | 1.134(0.651-1.974) | | 0.657 | |
| Smoking (Yes, No) | | 0.612(0.262-1.431) | | 0.258 | |  | | 0.701(0.315-1.561) | | 0.385 | |
| Alcohol use (Yes, No) | | 1.336(0.553-3.226) | | 0.520 | |  | | 1.198(0.517-2.776) | | 0.674 | |
| Tumor size (T3-T4, T1-T2) | | 0.597(0.213-1.677) | | 0.328 | |  | | 0.740(0.282-1.945) | | 0.541 | |
| Pathological grade (II-III, I) | | 2.042(1.209-3.448) | | **0.008** | |  | | 1.786(1.088-2.931) | | **0.022** | |
| Cervical nodal metastasis (N+, N0) | | 0.773(0.256-2.333) | | 0.648 | |  | | 0.961(0.339-2.720) | | 0.940 | |
| Clinical stage (III-IV, I-II) | | 1.428(0.412-4.948) | | 0.574 | |  | | 1.104(0.338-3.602) | | 0.870 | |
| NLR (≥2.9, <2.9) | | 1.982(1.150-3.416) | | **0.014** | |  | | 2.015(1.204-3.373) | | **0.008** | |
| **Combined cohort** | |  | |  | |  | |  | |  | |
| Age (>60, ≤60) | | 0.959(0.609-1.512) | | 0.857 | |  | | 1.018(0.674-1.537) | | 0.934 | |
| Gender (male, female) | | 1.224(0.752-1.993) | | 0.415 | |  | | 1.247(0.792-1.961) | | 0.340 | |
| Smoking (Yes, No) | | 0.447(0.222-0.902) | | **0.024** | |  | | 0.522(0.286-0.953) | | **0.034** | |
| Alcohol use (Yes, No) | | 1.258(0.608-2.604) | | 0.536 | |  | | 1.388(0.747-2.581) | | 0.300 | |
| Tumor size (T3-T4, T1-T2) | | 0.849(0.393-1.833) | | 0.676 | |  | | 0.833(0.416-1.665) | | 0.604 | |
| Pathological grade (II-III, I) | | 1.729(1.107-2.701) | | **0.016** | |  | | 1.455(0.972-2.178) | | 0.068 | |
| Cervical nodal metastasis (N+, N0) | | 1.039(0.464-2.327) | | 0.925 | |  | | 1.097(0.522-2.307) | | 0.807 | |
| Clinical stage (III-IV, I-II) | | 1.265(0.494-3.241) | | 0.624 | |  | | 1.321(0.564-3.093) | | 0.522 | |
| NLR (≥2.9, <2.9) | | 2.458(1.547-3.903) | | **<0.001** | |  | | 2.305(1.499-3.543) | | **<0.001** | |
| HR, hazard ratio; CI, confidence interval. | |  | |  | |  | |  | |  | |
