# Supplementary material for: Preoperative systemic immune-inflammation index predicts prognosis of patients with oral squamous cell carcinoma after curative resection
Source: J Transl Med. 2018 Dec 18;16:365. doi: 10.1186/s12967-018-1742-x (PMC6299596; doi:10.1186/s12967-018-1742-x)
Supplement: Supplementary file 8 — Additional file 8: Table S5. Multivariate survival analyses of prognostic factors associated with OS and DFS for OSCC. [file 12967_2018_1742_MOESM8_ESM.docx]

| **Additional Table S5. Multivariate survival analyses of prognostic factors associated with OS and DFS for OSCC** | | | | | | | | | | | |
| --- | --- | --- | --- | --- | --- | --- | --- | --- | --- | --- | --- |
| **Variables** | | **OS** | | | |  | | **DFS** | | | |
|  |  | **HR [95% CI]** | | ***P*** | |  | | **HR [95% CI]** | | ***P*** | |
| **Training cohort** | |  | |  | |  | |  | |  | |
| Age (>60, ≤60) | | 1.289(0.496-3.345) | | 0.602 | |  | | 1.522(0.672-3.448) | | 0.314 | |
| Gender (male, female) | | 1.857(0.666-5.176) | | 0.237 | |  | | 2.550(0.996-6.530) | | 0.051 | |
| Smoking (Yes, No) | | 0.177(0.039-0.811) | | **0.026** | |  | | 0.278(0.090-0.862) | | **0.027** | |
| Alcohol use (Yes, No) | | 2.331(0.460-11.800) | | 0.307 | |  | | 2.550(0.822-7.911) | | 0.105 | |
| Tumor size (T3-T4, T1-T2) | | 4.855(1.299-18.146) | | **0.019** | |  | | 2.188(0.757-6.325) | | 0.148 | |
| Pathological grade (II-III, I) | | 1.039(0.402-2.685) | | 0.938 | |  | | 1.063(0.490-2.309) | | 0.876 | |
| Cervical nodal metastasis (N+, N0) | | 2.498(0.652-9.564) | | 0.181 | |  | | 2.691(0.880-8.320) | | 0.083 | |
| Clinical stage (III-IV, I-II) | | 0.597(0.138-2.590) | | 0.491 | |  | | 1.065(0.306-3.713) | | 0.921 | |
| PLR (≥170.2, <170.2) | | 5.678(2.130-15.135) | | **0.001** | |  | | 3.318(1.414-7.788) | | **0.006** | |
| **Validation cohort** | |  | |  | |  | |  | |  | |
| Age (>60, ≤60) | | 0.884(0.523-1.492) | | 0.643 | |  | | 0.860(0.526-1.406) | | 0.549 | |
| Gender (male, female) | | 1.105(0.623-1.959) | | 0.732 | |  | | 1.072(0.621-1.851) | | 0.803 | |
| Smoking (Yes, No) | | 0.578(0.235-1.420) | | 0.232 | |  | | 0.667(0.285-1.561) | | 0.351 | |
| Alcohol use (Yes, No) | | 1.837(0.736-4.584) | | 0.192 | |  | | 1.565(0.653-3.751) | | 0.316 | |
| Tumor size (T3-T4, T1-T2) | | 0.652(0.234-1.6813) | | 0.412 | |  | | 0.814(0.313-2.115) | | 0.673 | |
| Pathological grade (II-III, I) | | 2.254(1.325-3.833) | | **0.003** | |  | | 1.952(1.183-3.222) | | **0.009** | |
| Cervical nodal metastasis (N+, N0) | | 0.863(0.288-2.584) | | 0.792 | |  | | 1.036(0.369-2.908) | | 0.947 | |
| Clinical stage (III-IV, I-II) | | 1.197(0.348-4.119) | | 0.775 | |  | | 0.952(0.294-3.077) | | 0.934 | |
| PLR (≥170.2, <170.2) | | 2.697(1.628-4.466) | | **<0.001** | |  | | 2.496(1.538-4.050) | | **<0.001** | |
| **Combined cohort** | |  | |  | |  | |  | |  | |
| Age (>60, ≤60) | | 0.966(0.616-1.514) | | 0.880 | |  | | 1.001(0.666-1.505) | | 0.995 | |
| Gender (male, female) | | 1.276(0.786-2.069) | | 0.324 | |  | | 1.315(0.838-2.064) | | 0.233 | |
| Smoking (Yes, No) | | 0.381(0.185-0.784) | | **0.009** | |  | | 0.454(0.245-0.841) | | **0.012** | |
| Alcohol use (Yes, No) | | 1.724(0.823-3.615) | | 0.149 | |  | | 1.803(0.964-3.375) | | 0.065 | |
| Tumor size (T3-T4, T1-T2) | | 1.139(0.550-2.360) | | 0.725 | |  | | 1.111(0.573-2.156) | | 0.755 | |
| Pathological grade (II-III, I) | | 1.731(1.108-2.705) | | **0.016** | |  | | 1.450(0.968-2.173) | | 0.072 | |
| Cervical nodal metastasis (N+, N0) | | 1.376(0.615-3.078) | | 0.438 | |  | | 1.414(0.674-2.967) | | 0.360 | |
| Clinical stage (III-IV, I-II) | | 0.933(0.376-2.316) | | 0.881 | |  | | 0.989(0.432-2.264) | | 0.979 | |
| PLR (≥170.2, <170.2) | | 3.203(2.071-4.954) | | **<0.001** | |  | | 2.600(1.727-3.912) | | **<0.001** | |
| HR, hazard ratio; CI, confidence interval. | |  | |  | |  | |  | |  | |
